# Knowledge, Attitude, and Practice Towards Child Immunisation Among Mothers Attending Magu District Hospital, Mwanza

**DOI:** 10.24248/eahrj.v8i1.755

**Published:** 2024-03-28

**Authors:** Pendo Ndaki, Madeline Kinyonga, Stanley Mwita

**Affiliations:** a Department of Biostatistics, Epidemiology and Behavioral Sciences, Catholic University of Health and Allied Sciences, Mwanza, Tanzania; b Department of Pharmaceutics and Pharmacy Practice, Catholic University of Health and Allied Sciences, Mwanza, Tanzania

## Abstract

**Background:**

Vaccines are administered to help the body develop immunity against a disease. A mother's understanding of the importance, safety, and benefits of vaccines can positively influence her decision to adhere to the recommended immunisation schedule. This study aimed to assess the knowledge, attitudes, and practices of mothers attending Magu District Hospital in Mwanza, Tanzania, towards child immunisation.

**Methods:**

A cross-sectional study was conducted among 216 mothers between April and May 2021. A convenient sampling technique was used to recruit mothers who consented to participate in this study. An interviewer-administered semi-structured questionnaire was used. The coded data were analysed using STATA Version 15.

**Results:**

About a quarter (27.3%) of respondents had good knowledge, while 64.8% showed positive attitudes towards child vaccination. Vaccine-preventable diseases that were commonly known by study participants were measles (90.7%) and poliomyelitis (81.9%). The majority of mothers (84.3%) would recommend others to vaccinate their children. About half of the children (50.9%) were fully immunized, while over a quarter (26.4%) of their children experienced side effects.

**Conclusion:**

The knowledge of mothers about vaccination was found to be inadequate, while the majority showed positive attitudes towards child immunisation. Only half of their children were fully immunized. The practice and knowledge of mothers on child immunisation should be enhanced by health education, awareness campaigns, and health promotion interventions.

## BACKGROUND

Vaccines are administered to help the body develop immunity against a disease.^1^ Child immunization is a cornerstone of public health interventions, contributing significantly to the reduction of morbidity and mortality among children worldwide.^2,3^ Immunization is one of public health's most successful and cost-effective interventions, saving up to three million lives annually.^4^ Immunization decreases the burden of infectious diseases and prevents illness, disability, and death from vaccine-preventable diseases, including diphtheria, pertussis, tetanus, measles, poliomyelitis, tuberculosis, and rubella.^5^ Globally, diphtheria, tetanus, poliomyelitis, tuberculosis, and pertussis vaccines have saved an estimated 400,000 lives.^6^ Measles vaccination resulted in a 79% reduction in worldwide measles deaths from 2000 to 2015.^7^

Tanzania's Expanded Program for Immunization (EPI) program provides free immunizations to Tanzania's full birth cohort of 2.2 million children.^8^ Tanzania, like many developing countries, has made progress in expanding its immunization coverage, but challenges remain in ensuring that every child receives the required vaccines.^9^ Approximately 20 million babies, the majority of whom reside in Sub-Saharan Africa, are either under or non-vaccinated. The coverage is 83% in Kenya, less than 80% in Uganda, and 86% on average worldwide.^10,11^ With the exception of vaccines administered beyond the age of one year, such as the measles-containing vaccine (2nd dose) and the human papillomavirus vaccine, Tanzania has been able to achieve and maintain high immunization coverage of 90% and above for most antigens for the past ten years.^12^

The knowledge, attitudes, and practices (KAP) of mothers, who are primary caregivers, play a critical role in determining the success of immunization programs.^13^ A mother's understanding of the importance, safety, and benefits of vaccines can positively influence her decision to adhere to the recommended immunization schedule.^14^ Conversely, misconceptions, concerns about vaccine safety, and limited awareness can lead to vaccine hesitancy or refusal.^15^ The negative attitudes of parents, such as the mother's fear of vaccination, adverse effects, and a tendency to ignore immunization because of mild illness, are considered barriers to a child's vaccination.^16^ Understanding the factors influencing maternal KAP towards child immunization is crucial for the improvement of immunization coverage and overall child health outcomes.

Previous studies done in Africa have been published regarding KAP in infant immunisations and reported diverse results.^17–19^ However, there is a scarcity of research focusing on maternal KAP towards child immunisation in Tanzania, which has outbreaks of vaccine-preventable diseases, especially measles,^20^ and no studies have been conducted in the Magu district. The hospital serves a diverse demographic of mothers and children from Magu, providing a representative sample for the study on child immunisation. Policymakers, program managers, and service providers will be able to identify the barriers to immunisation service uptake with help from the results of this study. Therefore, the aim of this study was to assess the knowledge, attitudes, and practices of mothers attending Magu District Hospital in Mwanza, Tanzania, towards child immunisation.

## METHODS

### Study Setting and Design

A descriptive, cross-sectional study took place in Magu district, Mwanza Region, between April and May 2021. Magu District, one of the seven districts comprising Mwanza Region, and part of the Lake Zone in Tanzania.^21^ Magu district hospital serves about 421,119 individuals.^22^

### Study Population

All mothers who visited the hospital with their children (under five years) were enrolled for interviews if they agreed to participate. Mothers with seriously sick children were excluded.

### Sample Size and Sampling Procedure

The Kish Leslie formula was used to calculate the sample size for this study.^23^ Using the prevalence of mothers with good immunisation knowledge from the Nigeria study, which was 17.0%,^17^ the minimum sample size was determined to be 216. A convenient sampling technique was used to recruit mothers who consented to participate in this study.

### Data Collection

The English semi structured questionnaire, which was translated into Swahili, sought data relating to the socio-demographic characteristics of mothers, knowledge, attitude, and practice toward child immunisation. Two fourth-year Catholic University of Health and Allied Sciences (CUHAS) bachelor of pharmacy students who served as research assistants collected the research data. The questionnaire used in this study was designed and modified following the method of previous studies.^17,24^ The sociodemographic information collected includes maternal age, education level, marital status, and number of children. Respondents were asked a total of 14 questions relating to their knowledge of immunisation. The total possible score was 14, as respondents received 1 mark for each correct answer, and no mark was awarded for wrong answers. The maximum score was 100%. Scores above 75% were considered good, between 50% and 75% were moderate, and less than 50% were considered poor.

The Attitude Likert scale of child vaccination consists of 10 statements with 3 points (agree), (uncertain), and (disagree). Scoring system: scoring was as follows: agree = 2, uncertain = 1, and disagree = 0. The total score of attitudes ranged from 0-20. Study participants who scored 50% and above on the attitude measuring item were considered to have a “positive attitude,” and those who scored below 50% were considered to have a “negative attitude.” The practice section involved the vaccination status of the child.

### Data Analysis

The coded data was entered into an Excel worksheet, cleaned, and then exported to STATA Version 15 for analysis. Data cleaning was performed to check for accuracy, consistencies, and missing values and variables. The results of the analysis were presented using frequency, percentages and, tables.

### Ethical Consideration

Ethical clearance for this study was sought from the CUHAS/BMC Research and Ethical Committee (IRB no. 1810/2021). Permission was requested from the district medical officer (DMO) in Magu and from the medical officer in charge at Magu District Hospital. Before the interview, written informed consent was signed by the participants who voluntarily agreed to participate in the study. To ensure confidentiality, no participant's name was recorded.

## RESULTS

### Socio-Demographic Characteristics

A total of 216 mothers were recruited into this study. The highest proportion of respondents, 97 (44.9%), were within the age group of 21–30 years. Almost three-quarters of the population, 161 (74.5%), had less than five children and were married, 163 (75.5%). Further, most respondents had primary education, 102 (47.2%). (Table 1).

**TABLE 1:** Social Demographic Characteristics of Study Participants (N=216)

Variable	Frequency	Percentage
Age (Years)		
≤20	40	18.5
21 - 30	97	44.9
31 - 40	62	28.7
≥41	17	7.9
Number of children		
1–4	161	74.5
>4	55	25.5
Marital Status		
Married	163	75.5
Unmarried	53	24.5
Education level		
Illiterate	30	13.9
Primary	102	47.2
Secondary	69	31.9
College and above	15	7.0

### Mothers' Knowledge on Child Immunisation

The majority of respondents were aware that immunisation is not for sick children only, 188 (87.0%); vaccination is important for children from the first day of birth, 183 (84.7%); vaccination prevents infectious diseases, 174 (80.5%); vaccination reduces death and disability, 187 (86.6%); and vaccination keeps children healthy, 192 (88.9%). Vaccine-preventable diseases that were commonly known by study participants were measles, 196 (90.7%); poliomyelitis, 177 (81.9%); diphtheria, tetanus, and pertussis, 159 (73.6%); tuberculosis, 153 (70.8%); and hepatitis B, 115 (53.2%). However, less than half of respondents, i.e., 98 (45.4%), agreed that child vaccinations prevent diarrhoea. Overall, 59 (27.3%) of respondents had good knowledge, 85 (39.4%) had moderate knowledge, and 72 (33.3%) had poor knowledge (Table 2).

**TABLE 2:** Mothers' Knowledge of Child Immunisation (N = 216)

Variables	Response	Frequency	Percentage
1. Immunizations for sick children only?	Yes	28	13.0
	No	188	87.0
2. Is vaccination important for children from the first day of birth?	Yes	183	84.7
	No	33	15.3
3. Does vaccination prevent infectious diseases?	Yes	174	80.5
	No	42	19.5
4. Does vaccination reduce death and disability?	Yes	187	86.6
	No	29	13.4
5. Can vaccination keep children healthy?	Yes	192	88.9
	No	24	11.1
6. Can diphtheria, tetanus, and pertussis be controlled through vaccinations?	Yes	159	73.6
	No	57	26.4
7. Can hepatitis B virus be prevented by vaccination?	Yes	115	53.2
	No	101	46.8
8. Can tuberculosis be prevented by vaccination?	Yes	153	70.8
	No	63	29.2
9. Can poliomyelitis be prevented by vaccination?	Yes	177	81.9
	No	39	18.1
10. Can child vaccinations control measles?	Yes	196	90.7
	No	20	9.3
11. Can child vaccinations prevent diarrhoea?	Yes	98	45.4
	No	118	54.6
12. Are malnutrition, low fever, and diarrhoea not contraindications to vaccination?	Yes	112	51.9
	No	104	48.1
13. Are some vaccinations related to fever and pain?	Yes	190	88.0
	No	26	12.0
14. Can vaccinations cause cramps and rashes?	Yes	104	48.1
	No	112	51.9
**Knowledge score**			
Poor		72	33.3
Moderate		85	39.4
Good		59	27.3

### Mothers' Attitude Towards Child Immunisation

One hundred and forty (64.8%) of respondents in this study showed positive attitudes towards child vaccination. Most of the respondents agreed that the completion of the immunization schedule is important, i.e., 193 (89.4%) and that vaccination maintains child health, 186 (86.1%). About 182 (84.3%) would recommend others vaccinate their children, while 176 (81.5%) agreed that vaccination is important. Almost half, 111 (51.4%) of mothers, agreed that the vaccines taken by infants are not too many (Table 3).

**TABLE 3:** Attitude of Mothers Towards Child Immunisation (N=216)

Attitude Factors	Respondents Categories		
	Disagree n (%)	Uncertain n (%)	Agree n (%)
1. Vaccination is important	13 (6.0)	27 (12.5)	176 (81.5)
2. Vaccination is safe	25 (11.6)	41 (19.0)	150 (69.4)
3. Vaccination maintains child health	6 (2.8)	24 (11.1)	186 (86.1)
4. Recommend others to vaccinate their children	11 (5.1)	23 (10.6)	182 (84.3)
5. Completion of Immunization schedule is important	5 (2.3)	18 (8.3)	193 (89.4)
6. Effective in prevention of infectious diseases	27 (12.5)	44 (20.4)	145 (67.1)
7. Vaccination reduces mortality rate	19 (8.8)	49 (22.7)	148 (68.5)
8. Vaccines do not have severe side effects	32 (14.8)	56 (25.9)	128 (59.2)
9. Infants cannot be infected with the disease they are immunized against.	18 (8.3)	41 (19.0)	157 (72.7)
10. The vaccines taken by infants are not too many.	58 (26.8)	47 (21.8)	111 (51.4)
**Attitude score n (%)**			
Positive	140 (64.8)		
Negative	76 (35.2)		

### Mothers' Practice Toward Child Immunisation

The majority of mothers had vaccination cards, 202 (93.5%). About half of the children, i.e., 110 (50.9%), were fully immunised, while almost a quarter i.e., 57 (26.4%), experienced side effects. Sixty-five children (30.1%) received antipyretics at the immunization site after vaccination (Table 4).

**TABLE 4:** Mothers' Practice Toward Child Immunisation (N = 216)

Variables	Response	Frequency	Percentage
Vaccination card	Available	202	93.5
	Unavailable	14	6.5
Vaccination status	Fully immunised	110	50.9
	Incompletely immunized	97	44.9
	Not immunized at all	9	4.2
Child experienced any vaccination side effect	Yes	57	26.4
	No	159	73.6
Received anti-pyretic after vaccination	Yes	65	30.1
	No	151	69.9

## DISCUSSION

To reduce the morbidity and mortality associated with infectious diseases, immunization is a crucial public health intervention. Vaccinations are one of the easiest, safest, and most economical ways to save and enhance the lives of children throughout the world.^25^ Vaccinating children against diseases that can be prevented by vaccines is crucial for reducing poverty, boosting productivity, and saving lives.^26^ Mothers' pre-existing knowledge and positive attitude are crucial for children's vaccination outcomes.^27^ The current study revealed that about a quarter of mothers had good knowledge regarding child vaccinations, and almost two-thirds had a positive attitude, while about half of children were fully immunized.

In agreement with this study, previous studies^28–30^ also reported inadequate mothers' knowledge about child immunization. This highlights the need for targeted educational interventions to improve awareness about the importance of immunization, particularly in addressing gaps in understanding. The majority of mothers in this study were aware that immunization is not solely for sick children, emphasizing the importance of vaccination as a preventive measure. Further, they mentioned that the primary goal of immunization is to prevent infectious diseases and reduce death and disability. These findings are in line with the previous studies conducted in Saudi Arabia, which revealed that 90.1% of mothers agreed that vaccination prevents infectious diseases, while 93.9% agreed that vaccination reduces death and disability.^24^ Consistent with previous studies,^17,24,29^ common vaccine-preventable diseases known to the current study participants were measles and poliomyelitis. These findings could have been attributed to extensive public health campaigns that often focus on polio and measles and high incidences in the past that caused significant morbidity and mortality.^31^

The positive attitudes displayed by the majority of mothers toward child immunization are promising for the success of immunization programs. A significant proportion expressed a positive attitude, which can have a considerable impact on their willingness to engage in the immunization process for their children. This proportion is higher compared to the study findings from Egypt^30^ and Ethiopia.^18^ This might be due to the community's increased interest in getting information about vaccines and vaccination-preventable diseases from healthcare professionals and the media to improve their awareness, which in turn changes mothers' attitudes toward the importance of immunisation. The high percentage of mothers who would recommend vaccination to others indicates a potential for positive peer influence within communities.

The current study revealed that only half of children were fully immunized; thus, there is a need for improvement in terms of adherence to the recommended immunisation schedule. This finding is higher than the results reported from Sudan (42.9%)^30^ and India (34.8%).^32^ On the contrary, the study conducted in Georgia reported a higher proportion of fully immunized children (64.0%).^33^ The inconsistency may be due to differences in socio-demographic characteristics and study settings. Almost a quarter of children experienced vaccination side effects. This is consistent with the study done in Sudan^30^ and lower than the other study conducted in the United Arab Emirates.^34^ Experiencing side effects may be a cause for concern, and addressing these concerns and providing proper information to parents can help alleviate any fears or misconceptions related to immunisation side effects. Incomplete immunisation could be attributed to long waiting times at health institutions, inadequate information about the vaccination schedule, and a fear of side effects after vaccination.^18,33^

### Limitations

This study is limited by the fact that it cannot show a cause-and-effect relationship since it is cross-sectional. Another limitation of this study is the use of a questionnaire; hence, it is difficult to ensure that respondents gave honest responses. Further, it was an institution-based study conducted in one hospital, thus its inability to represent the whole population. Lastly, this study did not report the causes of negative or positive practices. However, the findings of this study provide the basis for future studies to evaluate factors affecting the vaccination status of children in similar settings.

## CONCLUSION

The knowledge of our mothers about vaccination was found to be inadequate, while the majority showed positive attitudes towards child immunisation. Only half of their children were fully immunized. The practice and knowledge of mothers on child immunisation should be enhanced by health education, awareness campaigns, and health promotion interventions. It's recommended to engage the community in the promotion of child vaccination by involving community organizations, local authorities, and healthcare workers. Programs for community outreach should be utilized to promote vaccine uptake and disseminate accurate information.
